# Pangenome Reconstruction of *Mycobacterium tuberculosis* as a Guide to Reveal Genomic Features Associated with Strain Clinical Phenotype

**DOI:** 10.3390/microorganisms11061495

**Published:** 2023-06-04

**Authors:** Andrea Monserrat Negrete-Paz, Gerardo Vázquez-Marrufo, Ana Gutiérrez-Moraga, Ma. Soledad Vázquez-Garcidueñas

**Affiliations:** 1 División de Estudios de Posgrado, Facultad de Ciencias Médicas y Biológicas “Dr. Ignacio Chávez”, Universidad Michoacana de San Nicolás de Hidalgo, Morelia 58020, Michoacán, Mexico; andrea.negrete@umich.mx; 2 Centro Multidisciplinario de Estudios en Biotecnología, Facultad de Medicina Veterinaria y Zootecnia, Universidad Michoacana de San Nicolás de Hidalgo, Tarímbaro 58893, Michoacán, Mexico; gvazquez@umich.mx; 3 Instituto de Ciencias Biomédicas, Vicerrectoría de Investigación y Doctorados, Universidad Autónoma de Chile, Santiago 7500912, Chile; ana.gutierrez@uautonoma.cl

**Keywords:** pangenome, tuberculosis, extrapulmonary

## Abstract

Tuberculosis (TB) is one of the leading causes of human deaths worldwide caused by infectious diseases. TB infection by *Mycobacterium tuberculosis* can occur in the lungs, causing pulmonary tuberculosis (PTB), or in any other organ of the body, resulting in extrapulmonary tuberculosis (EPTB). There is no consensus on the genetic determinants of this pathogen that may contribute to EPTB. In this study, we constructed the *M*. *tuberculosis* pangenome and used it as a tool to seek genomic signatures associated with the clinical presentation of TB based on its accessory genome differences. The analysis carried out in the present study includes the raw reads of 490 *M. tuberculosis* genomes (PTB *n* = 245, EPTB *n* = 245) retrieved from public databases that were assembled, as well as ten genomes from Mexican strains (PTB *n* = 5, EPTB *n* = 5) that were sequenced and assembled. All genomes were annotated and then used to construct the pangenome with Roary and Panaroo. The pangenome obtained using Roary consisted of 2231 core genes and 3729 accessory genes. On the other hand, the pangenome resulting from Panaroo consisted of 2130 core genes and 5598 accessory genes. Associations between the distribution of accessory genes and the PTB/EPTB phenotypes were examined using the Scoary and Pyseer tools. Both tools found a significant association between the *hspR*, *plcD*, *Rv2550c*, *pe_pgrs5*, *pe_pgrs25*, and *pe_pgrs57* genes and the PTB genotype. In contrast, the deletion of the *aceA*, *esxR*, *plcA*, and *ppe50* genes was significantly associated with the EPTB phenotype. *Rv1759c* and *Rv3740* were found to be associated with the PTB phenotype according to Scoary; however, these associations were not observed when using Pyseer. The robustness of the constructed pangenome and the gene–phenotype associations is supported by several factors, including the analysis of a large number of genomes, the inclusion of the same number of PTB/EPTB genomes, and the reproducibility of results thanks to the different bioinformatic tools used. Such characteristics surpass most of previous *M. tuberculosis* pangenomes. Thus, it can be inferred that the deletion of these genes can lead to changes in the processes involved in stress response and fatty acid metabolism, conferring phenotypic advantages associated with pulmonary or extrapulmonary presentation of TB. This study represents the first attempt to use the pangenome to seek gene–phenotype associations in *M. tuberculosis*.

## 1. Introduction

Tuberculosis (TB) affects more than 14 million people annually, and it is estimated that 2 billion people are currently infected with *Mycobacterium tuberculosis* (MTB), the causal agent of TB. The main causes attributed to the rising number of TB cases are coinfection with the human immunodeficiency virus (HIV), the emergence of resistant and multi-resistant MTB strains, and the low efficacy of the currently used vaccine [1]. Although it is estimated that one-third of the world’s population is infected with MTB, only between 5 and 10% of cases will develop the disease, either in the first or second year after infection (primary TB) or due to the reactivation of infection throughout life (secondary TB). In the rest of the cases, the infection remains latent throughout life [1]. In latent TB, bacillary replication is absent or below some undefined threshold as a result of immunologic control. Consequently, no evidence of clinical TB is exhibited [2]. Namely, it is a state of persistent immune response to stimulation by MTB antigens without signs or symptoms of disease. TB is limited to the lungs in approximately 85% of cases, while the remaining percentage develops extrapulmonary tuberculosis (EPTB). Among EPTB cases, miliary and meningeal TB are considered the most severe forms of the disease, generally associated with a poor prognosis [3].

The wide diversity in clinical manifestations and disease severity is one of the most intriguing aspects of TB. This diversity has been primarily attributed to host factors, such as HIV infection, the presence of diabetes, smoking, age, and malnutrition, among others. However, host factors do not fully explain such variations, and a large number of epidemiological and experimental studies have shown that the genetic diversity of MTB strains also contributes to it [4]. Some of these studies conduct a whole genome comparative analysis, which is a powerful tool that allows for comparisons between pulmonary (PTB) and EPTB strains in the search for subtle genetic determinants associated with tropism. This approach was used to compare 8 EPTB with 13 local PTB strains from Malaysian patients. The study identified various rearrangements, including translocations, inversions, insertions, and deletions, as well as non-synonymous SNPs in the cerebrospinal fluid isolated strains that were not observed in the genomes of the PTB strains [5]. The rearrangements are located in a large number of genes coding for PE (proline–glutamate)/PPE (proline–proline–glutamate) proteins, as well as for transcriptional and membrane proteins. Additionally, most of the common SNPs in the genomes of meningeal strains were observed in genes encoding PE and PPE proteins. However, it was not possible to identify genetic determinants or variations that were common to all the EPTB strains. Similarly, in India, five strains from EPTB patients belonging to lineage 3 exhibited 279 exclusive genomic variations that were not present in PTB strains. However, these variations were not shared by all strains within the same clinical phenotype [6].

Recently, two independent studies compared the genomes of a large number of EPTB and PTB strains isolated from Indonesia and Thailand. The first one analyzed 322 strains, consisting of 106 EPTB and 206 PTB strains [7], whereas the second one examined 293 strains, including 73 EPTB and 220 PTB strains [8]. These studies used different methodological strategies to search for genetic determinants associated with meningeal TB. Although both studies identified exclusive genomic variants shared by most of the genomes of the EPTB strains, these variants were not consistently found among such strains. Possible reasons for such inconsistency could be (a) the low number of EPTB strains used in the analysis; (b) the strains analyzed belong to a single geographic region, causing a bias associated with the predominance of a specific lineage; and (c) the strategy used for the genomic analysis. Consequently, to date, there is no consensus on the genetic determinants that contribute to extrapulmonary forms of TB.

The use of comparative genomic analysis, derived from the construction of the pangenome, may represent a novel strategy for identifying differences that could explain the clinical presentation of the disease. Pangenome construction refers to the collection of all genes present in a particular species or group of microorganisms, encompassing both a central or core genome and an accessory genome. The core genome encompasses the genes that are present in all strains, whereas the accessory genome comprises dispensable genes that can be found in two or more strains, as well as genes that are unique to a single strain [9]. Within the pangenome, the accessory genome is believed to have a significant impact on phenotypic variation and genome evolution [10]. Although dispensable genes and strain-specific genes are considered secondary elements that determine strain-specific characteristics and are not essential for survival, they also play a crucial role in bacterial adaptation to specific environments [11]. Several previous studies have focused on the *M. tuberculosis* pangenome [10,11,12,13,14,15,16]. However, these studies do not consider the use of the pangenome as a tool to compare or differentiate between PTB and EPTB strains. Therefore, this study aims to evaluate the composition of the accessory genome of the pangenome constructed from PTB and EPTB genomes in order to identify genomic signatures associated with phenotypic variation in relation to the clinical presentation of TB.

## 2. Materials and Methods

### 2.1. WGS Data Retrieval

A total of 490 previously collected sequence read archives of MTB [17] were used in this study, comprising 245 PTB strains and 245 EPTB strains. The number of genomes included in the construction of the pangenome was limited by the availability of EPTB genomes in public databases. All EPTB strains deposited in NCBI-SRA at the time of this study that met the following criteria were selected: (1) being isolated from cases in which the reported site of infection was not pulmonary or miliary, (2) negative HIV status, and (3) availability of associated metadata, including country of isolation and absence of comorbidities. Additionally, 245 *M. tuberculosis* genomes that caused PTB were obtained, following the inclusion criteria of country of isolation, absence of HIV infection, and no reported comorbidities in the metadata. This resulted in a total of 490 raw datasets of genome sequence reads. Furthermore, ten *M. tuberculosis* strains were collected from patients with confirmed diagnoses of PTB (*n* = 5) and EPTB (*n* = 5) by the Tuberculosis Program of the Health Secretary of the Michoacán State, Mexico (Table S1, MYC strains). The genomes of these strains were sequenced by the GeneWiz sequencing center (South Plainfield, NJ, USA) using Illumina Next-Generation HiSeq Sequencing (Illumina, San Diego, CA, USA).

### 2.2. Genome Assembly and Annotations

Fastq reads underwent a quality control analysis using FastQC (http://www.bioinformatics.babraham.ac.uk/projects/fastqc, last accessed on 15 August 2022) and were subsequently filtered using Trim Galore (http://www.bioinformatics.babraham.ac.uk/projects/trim_galore/, last accessed on 21 October 2022). Genome assembly was conducted using SPAdes [18]. To enhance the quality of the genomes, contigs shorter than 500 bp long were excluded from the assemblies (Table S1). The quality assessment of the assemblies was conducted using QUAST [19], and the resulting scaffolds were annotated using Prokka [20]. The latter software package allows for protein annotation by utilizing sequence similarity to other proteins in various databases. Coverage of the reference genome was achieved by aligning all the selected strains to the complete reference genome of the H37Rv strain (NC_000962.3).

### 2.3. Pangenome Construction and Analysis

The pangenome analysis was performed using Roary v3.13.0 [21], utilizing the GFF3 files generated by Prokka. This analysis was conducted with the following parameters: 90% identity threshold for BLASTP, a set number of clusters at 100,000, and splitting paralogs. Additionally, Panaroo v1.3.3 was employed in strict mode [22] to assess the size of the core and accessory genome. The search for accessory genes exclusive to the EPTB or PTB phenotypes was performed using the query_pan_genome option (-difference), which allows for the identification of differences in gene content between two specific groups. Associations between the distribution of accessory genes and the PTB or EPTB phenotype were made using Scoary [23], which utilizes the Roary gene_presence_absence.csv output files to perform a genomic association analysis. Scoary uses the output from Roary (gene_presence_absence.csv) and trait.csv for the genotype and phenotype input files, respectively. Each accessory gene in the pangenome was scored according to its potential correlation with the PTB or EPTB phenotype using a 2 × 2 contingency table. A Fisher’s exact test was performed, and the Benjamini–Hochberg false discovery rate (FDR) adjustment was applied using Scoary to correct for multiple comparisons. Another recently developed tool was employed to identify the MTB genes that are associated with EPTB and PTB phenotypes. This bacterial genome-wide association study (GWAS) was conducted using Pyseer version 1.3.10 [24] since it incorporates a correction for population structure. It is essential for microbial GWAS methods to apply a correction for population structure in each test, although the specifics of this correction may vary depending on the methods and data used. In some cases, further adjustments may be necessary if confounding factors are genetically stratified as a result of the sampling strategy [25]. The cut-off for a significant association in both analyses was set at a *p* value lower than 0.05.

### 2.4. Phylogenetic Analysis

As part of the Roary output, we generated a tree based on the presence and absence of accessory genes, which was visualized using FastTree 2 [26]. This accessory binary tree was annotated in iTOL [27] in order to identify similarities in the composition of the accessory genome between PTB and EPTB strains. The lineage and sublineage of MTB strains were determined using the MTBSeq tool [28].

### 2.5. Statistical Analysis

Fisher’s exact test was applied to the query_pan_genome data obtained using R, with a statistical significance threshold of *p* < 0.05.

## 3. Results

**Figure 1:**
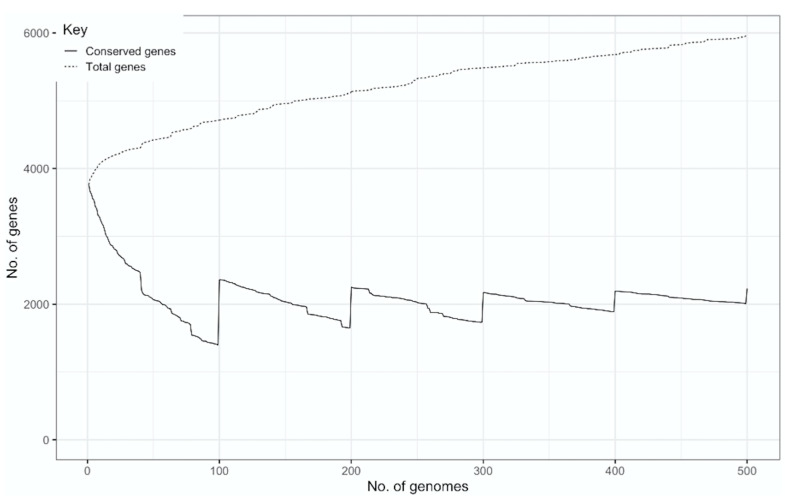
Pangenome plot of the global gene repertoire of *M. tuberculosis*. It is observed that, as more genomes are added, the total number of genes does not increase significantly.

We performed de novo sequence assembly using the SPAdes genome assembler [18]. The high quality of the genome assemblies (Table S1) enables accurate gene prediction using Prokka. The quality parameters of an assembly, such as a high genome fraction covered, a low number of contigs, and a genome length consistent with the analyzed species, are considered crucial to ensure the suitability of these assemblies for gene identification analysis [29]. The 500 strains selected for this study had an average of 115 contigs (>500 bp) and a mean genome size of 4,491,845 bp. The reference genome coverage was 95.06%, and the average GC% was 66. After filtering out genomes with poor quality, the construction of the pangenome enabled us to identify 5961 gene clusters using Roary and 7728 gene clusters using Panaroo. This constructed pangenome exhibits an open pangenome, as can be seen in Figure 1. 

**Figure 2:**
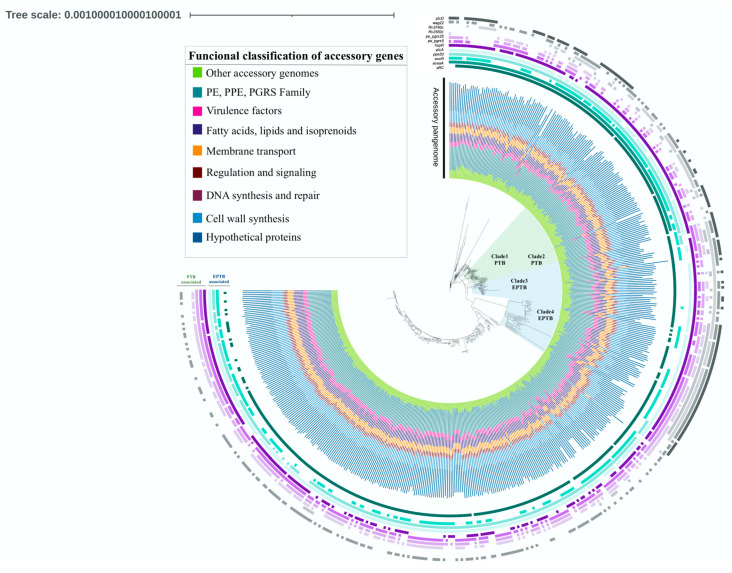
Phylogenetic construction of the analyzed *M. tuberculosis* strains showing the functional classification of the accessory pangenome. The light-green clades 1 and 2 exclusively contain PTB strains (*n* = 57), while the light-blue clades 3 and 4 consist solely of EPTB strains (*n* = 72). The presence of EPTB-associated genes (*aftc*, *aceaA*, *plcA*, and *esxR*) and PTB-associated genes (*hspR*, *plcD*, *pe_pgrs5*, *pe_pgrs25*, *pe_pgrs57*, *Rv3740c*, *Rv2550c*, and *Rv1759c*) are shown.

**Figure 3:**
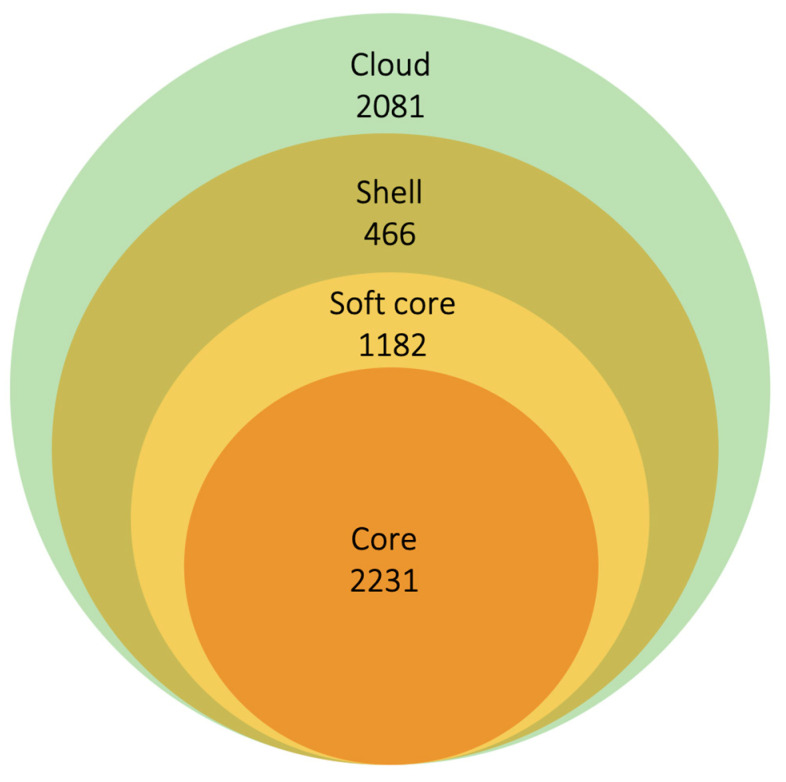
Composition of the PTB/EPTB *M. tuberculosis* Roary pangenome. The analysis of the pangenome allows classifying the gene content into four large groups: (1) core genes, present in 100% of the genomes, (2) soft-core genes, found in 95 to 99% of the genomes, (3) shell genes, observed in 15 to 95% of the included genomes, and (4) cloud genes, present in one to 15% of the genomes.

Figure 2 displays the tree generated via Roary, which is based on the presence and absence of accessory genes. The annotated tree revealed four distinct clades, each exclusively composed of strains belonging to a single clinical phenotype. The two green-light clades exclusively contain PTB strains (Clade1 PTB, *n* = 44; Clade2 PTB, *n* = 13), whereas the two blue-light clades exclusively consist of EPTB strains (Clade3 EPTB, *n* = 38; Clade4 EPTB, *n* = 34). The analysis of the pangenome allows for the classification of gene content into four large groups: (1) core genes, present in 100% of the genomes, (2) soft-core genes, found in 95 to 99% of the genomes, (3) shell genes, observed in 15 to 95% of the included genomes, and (4) cloud genes, present in 1 to 15% of the genomes [21]. After the analysis with Roary, 2231 core genes, 1182 soft-core genes, 466 shell genes, and 2081 cloud genes were identified (Figure 3). The analysis performed with Panaroo showed a pangenome integrated by 7728 genes, with 2130 genes belonging to the core genome and 5598 to the accessory genome. As expected, the genes in the core genome are similar to those obtained using Roary (2231 genes). However, the accessory genome identified by the latter tool contains fewer genes (3729 genes) than the one obtained using the former tool. Previous studies have reported that Roary often generates larger accessory genomes than similar bioinformatic tools [22]; however, this was not observed in our study.

Of the 3729 genes comprising the Roary accessory genome, 29.3% correspond to hypothetical proteins or proteins of unknown function, 25% belong to the PE, PPE, and PGRS family, 13% are genes coding for fatty acids, lipids, and isoprenoids synthesis, 11.8% of the genes participate in cell wall synthesis, 6.6% are involved in membrane transport, 4.6% are considered virulence factors, 3.6% are involved in regulation and cell signaling, 1.4% play a role in DNA synthesis and repair, and 8.5% of the accessory genes fall into other functional categories observed in smaller proportions (Figure 2).

In the search for exclusive genes among strains belonging to the EPTB phenotype, two genes were identified exclusively in the genomes of these strains: *pstAq1* (phosphate transporter protein) and a gene coding a hypothetical protein. However, it was not possible to establish a statistical association due to the low proportion of these genes in the genomes of the EPTB strains (<15). A similar phenomenon was observed in PTB strains, where genes encoding the DevS1 (histidine kinase regulator) and Mtr1 (mycothiol reductase) proteins were identified as exclusive of this phenotype. Likewise, it was impossible to establish a statistical association as they are present in low proportions in the genomes of the PTB strains. Further, differences in gene distribution across both genotypes were identified. The performed analysis also demonstrates an association of the PTB phenotype with the deletion of *hspR*, *plcD*, and *Rv2550c*, which are classified as virulence genes, and with *pe_pgrs5*, *pe_pgrs25*, *Rv1759c*, *pe_pgrs57*, and *Rv3740c* genes. Contrastingly, the deletion of *aceA*, *esxR*, *plcA*, and *ppe50* genes is associated with the EPTB phenotype (Table 1). The Pyseer analysis confirmed the previously obtained results with Scoary, demonstrating that *aftC*, *aceaA*, *plcA*, *esxR*, and *ppe50* genes are associated with the EPTB phenotype. Although the *p*-values varied, the association remained significant even after correction for population structure (Table S2). For the PTB phenotype, an association was found with *hspR*, *plcD*, *vapB20*, *pe_pgrs5*, *pe_pgrs25*, and *pe_pgrs57* genes. In this analysis, the genes *Rv1759c* and *Rv3740c* were not found to be associated with the PTB phenotype. However, such an association was found in the previous analysis using Scoary, which could be attributed to the population correction applied.

Regarding the genotyping classification, the sublineages included in PTB clades 1 and 2 were 4.3.1, 4.3.2, 4.3.3, 4.3.4.2, and 4.5 (Table S3). All these sublineages, except 4.5, belong to the Latin American Mediterranean family (LAM), which has been previously associated with the PTB phenotype [17]. The strains grouped in EPTB Clade3 and 4 belong to different sublineages (Table S4), of which 4.2.1.1, 1.1.1, and 1.2.1 have previously been associated with the EPTB phenotype [17]. In general, the PTB and EPTB exclusive clades exhibit a similar distribution of the accessory genome and gene deletion, as can be seen in Figure 2. This highlights the importance of the accessory genome in determining the clinical phenotype in TB.

## 4. Discussion

Multiple genomic studies have attempted to identify genetic determinants in MTB strains that may help explain the clinical TB phenotype. However, the identified genomic variations have not consistently been found among the strains corresponding to PTB or EPTB phenotypes. The present work approaches this issue from a different perspective than previous studies, i.e., through the construction of the *M. tuberculosis* pangenome. To the best of our knowledge, this study uses the largest number of genomes from EPTB strains analyzed to date and represents the first analysis that considers a global collection of strains from this clinical phenotype. The inclusion of global strains in this study contributes to increasing the availability of EPTB strains isolated from Latin America, particularly from Mexico, an underrepresented country in databases concerning MTB genomes, and especially in relation to the EPTB phenotype. At the time of publication of the present paper, only one MTB genome assembly isolated from Latin America from a patient with meningeal TB is available [30], which highlights the need to increase the number of EPTB strains sequenced in this region. 

The Roary analysis performed in this study with the included genomes reveals that the *M. tuberculosis* pangenome is open, suggesting that the number of accessory genes will continue to increase. A pangenome is considered almost closed when the curve representing the total number of genes (pangenome curve) ceases to significantly increase with the addition of new genomes and starts to flatten. This trend does not match our results, as illustrated in Figure 1. As in the present work, several studies have previously reported *M. tuberculosis* pangenomes to be open [11,12,16], but there are also others that report closed pangenomes [10,15]. Each of these previous reports, as well as the present work, used different bioinformatic tools and homology criteria/cutoff to reconstruct the *M. tuberculosis* pangenome, which can partially explain such a discrepancy (Table 2). This can also explain the differences in pangenome structure between studies. As stated in the Materials and Methods section, the number of genomes included in the reconstruction of the *M. tuberculosis* pangenome was limited by the availability of EPTB genomes and metadata of interest for the analysis. Despite this, to the best of our knowledge, this study outscores by between three- to ten-times the number of *M. tuberculosis* genomes in most but one of the previous pangenome studies (Table 2). Thus, considering the large number of global strains analyzed in this study and the robust bioinformatic analysis performed, it can be suggested that despite its proposed low genome variation, *M. tuberculosis* still retains the capability to add genes to its global gene repertoire. Gene duplication and fission are likely the mechanisms involved in the acquisition of new genes by *M. tuberculosis* [31]. 

The absence of exclusive genes in a specific phenotype can be attributed to the high degree of conservation observed in MTB genomes. It has been established that the overall level of sequence variation in MTB is low [32]. This low genetic variability is partly due to the null acquisition of genetic material through horizontal transfer. It is noteworthy that the level of genetic diversity within the *M. tuberculosis* complex has been a subject of debate. Early gene-specific studies revealed low DNA sequence diversity and led to the notion that strain variability in MTB was insignificant and without clinical importance [33]. However, further studies using a larger number of markers demonstrated the presence of genetic variability between MTB strains isolated from different geographical regions [34,35]. Recent comparative genomic analyses based on whole-genome sequencing have revealed differences, even between two strains that differ by up to 2000 single nucleotide polymorphisms (SNPs), a genetic distance comparable to the distance between the *M. tuberculosis* and *M. bovis* species [36]. Although this diversity is substantially less than that seen in other Gram-positive human pathogenic bacterial species, there is a growing body of evidence showing that these relatively subtle differences may have functional and clinical consequences [37].

**Table 2:** Comparison between *Mycobacterium tuberculosis* pangenome studies ^1^.

Analyzed Genomes	Core Genome	Accessory Genome Genes	Pangenome Open/Closed	Bioinformatic Tools	Reference
36	3679	2086	Open	PGAP pipeline	[11]
47	3650	1196	Open	BLASTP, GET_HOMOLOGUES, Perl script	[16]
96	2066	6033	Open	BLAST2GO and Perl scripts	[12]
145	3736	708	NA ^4^	Roary	[38]
150	1251	3758	Nearly closed	BPGA tool	[15]
183 ^2^	1166	5870	Closed	BLASTPBLOSUM62	[10]
1595 ^3^	3419	7620	Closed	CD-hit package v4.6	[14]
500	2231	3729	Open	Roary	This study

^1^ Only core and accessory genes were included despite further soft, cloud, and shell subdivisions described in studies similar to this one. ^2^ This study analyses the genomes of other members of the *Mycobacterium tuberculosis* complex, including *M. tuberculosis* var. *africanum*, *M. tuberculosis* var. *bovis*, and BCG (Bacillus Calmette-Guérin) strains. ^3^ In this study, the PE/PPE protein coding genes were excluded from the pangenome analysis; ^4^ NA, not assessed.

The analysis conducted in this study showed a statistically significant association between the presence of the *aftC* gene and the EPTB phenotype. Genetic recombination processes may be responsible for the observed differences in the gene content shared by the EPTB and PTB phenotypes, as observed in the case of the *aftC* gene. The AftC protein is involved in the metabolism of arabinan, which serves as a precursor for the synthesis of arabinogalactan, one of the key polysaccharides present in the mycobacterial cell wall [39]. Therefore, the deletion of the *aftC* gene in PTB strains could result in a decrease in the content of arabinogalactans in the mycobacterial cell wall, leading to a reduction in mycolic acid levels. This biochemical event chain can potentially cause decreased permeability and reduced stress resistance in these strains, preventing their migration to other parts of the body.

In a previously constructed pangenome with 183 MTB strains, 20% of the MTB virulence genes were found in the core genome, and *plcA*, *plcD*, and *aceA* genes were found in the remaining 80% of the accessory genome [10], as observed in our study. It has been stated that MTB can trigger a change between active and dormant stages based on the availability of carbon sources by alternating metabolic pathways. One of the genes involved in this process is *aceA*, which codes for an isocitrate lyase [40]. It has been suggested that under hypoxic conditions where lactate and pyruvate synthesis is more likely to occur, β-oxidation and glyoxylate shunt involving isocitrate lyase gene pathways are actively involved in fatty acid degradation [41]. In this regard, MTB has four genes (*plcABCD*) involved in the process of degrading phospholipids of the host cell membrane. This degradation releases fatty acids that can be utilized by the pathogen in the β-oxidation cycle and the glyoxylate shunt [41]. Whereas *plcABC* are full-length genes, *plcD* is truncated and located in a different locus of the MTB genome. It can be hypothesized that the deletion of *plcA* and *aceaA* genes in EPTB strains could lead to low fatty acid availability as a source of energy for latent MTB, preventing the pathogen from entering a state of dormancy and, thus, promoting its extrapulmonary spread. On the other hand, fatty acids are the primary energy source for MTB during the persistence phase, probably obtained via triacylglycerol. *Rv3740c* is a gene that is predicted to encode a triacylglycerol synthase. Furthermore, putative triacylglycerol synthase genes are known to be induced when the pathogen enters a hypoxic state and in response to NO treatment [42]. The absence of *Rv3740c* is probably compensated for by the presence of other proteins that provide fatty acids to MTB during the persistent phase, such as those coding for *aceaA* and *plcA* present in PTB strains.

Iron is an essential micronutrient for MTB growth but is very limited within the human host. To ensure proper iron uptake, MTB secretes siderophores, small molecules with a high affinity for iron, and then picks up iron-loaded mycobactins and carboxymycobactins from the environment [43]. It has been established that the ESX-3 secretion system is essential for siderophore-mediated iron uptake and for adapting to low zinc availability. The EsxG and EsxH proteins form a heterodimer that is secreted by the ESX-3 secretory system. This system is not only essential for bacterial metabolism but plays a significant role in immunological activity by inhibiting phagosome maturation [44]. A recent study showed that strains with a deletion of the *esxG* or *esxH* gene regained their ability to grow in the absence of iron after infection in mice [45]. This ability was conferred by mechanisms that caused overexpression of an ESX-3 paralogous region that lacks genes for the secretion apparatus but encodes EsxR and EsxS. Even though EsxR is a protein with an unknown function, all of these findings suggest the possibility that EsxR could be involved in the process of iron uptake and in the elucidated immunological response. The EsxG-EsxH heterodimer facilitates the secretion of several members of the proline–glutamic acid (PE) and proline–proline–glutamic acid (PPE) protein families [46]. In this work, the gene coding for the PPE50 protein belonging to this family was associated with EPTB. It is known that a deletion of the ppe50/ppe51 region can increase tolerance to isoniazid and rifampicin, a resistance mechanism that is probably related to carbon metabolism, specifically glycerol uptake [47]. Furthermore, a partial deletion of this gene, which has been previously associated with the EPTB phenotype [17], has been reported in Nonthaburi genotype strains isolated from patients with meningeal TB [48]. Interestingly, all the strains grouped in the exclusive EPTB clades exhibit a deletion of *ppe50* and *aceaA*, regardless of their sublineage classification. These findings suggest the potential role of these deletions in conferring pathogenicity advantages.

According to PTB gene associations, *hspR* was found to be associated with the pulmonary presentation of the disease. The HspR protein is a transcriptional modulator that represses the transcription of genes encoding heat shock proteins [49]. It has been shown that the deletion of the *hspR* gene facilitates the early stages of pulmonary infection by the bacteria [49]. However, during the chronic phase, it leads to increased clearance of MTB [50], thereby reducing the bacteria’s ability to spread throughout the body. The *Rv2550c* gene encodes the VapB20 protein, which is a component of the *M. tuberculosis* VapBC20 toxin–antitoxin system. Under normal conditions, VapB and VapC interact to form a hetero-octameric complex that inhibits their own expression [51]. Under stress conditions, VapB antitoxin is degraded by cellular proteases to release a free VapC toxin, inhibiting cell growth. PTB strains that do not have the Rv2550c gene may exhibit a distinct phenotype, characterized by slowed or completely inhibited cell growth, impairing its possibilities to escape from the lungs. The function of the *plcD* is still a subject of controversy, but there have been suggestions that its interruption could contribute to the development of extra-thoracic TB [52]. However, in the present study, we found a statistically significant association between the absence of *plcD* and the PTB phenotype. The *plcD* region is widely recognized as a favored insertion site for the IS6110 insertion sequence, which has the potential to cause gene deletions [53]. The reason why this gene deletion is observed in PTB strains more frequently remains unknown, and future research is needed in order to clarify this.

The PE, PPE, and PGRS family accounts for about 10% of the coding capacity of the MTB genome [54]. Many proteins of this family are localized at the cell surface, being attached to the membrane, while other members are involved in modulating the host immune response and virulence [55]. In this work, the *pe_pgrs5*, *pe_pgrs25*, *pe_pgrs57*, and *Rv1759c* genes were found to be associated with the PTB phenotype. The proteins encoded by these genes have diverse functions. For example, the *Rv1759c* gene encodes the Wag22 antigen of the pe_pgrs family, which possess fibronectin activity and is involved in the maintenance of latent infection in a chronic infection model [56], inducing the production of high levels of interferon gamma. PE_PGRS5 promotes alterations to intracellular calcium homeostasis, resulting in an increase in the production of NO and reactive oxygen species and inducing caspase-8-mediated apoptosis [57]. It has been shown that PE_PGRS5 is expressed during MTB infection at the later stages of lung granulomas, suggesting that the bacterium employs this protein as a dissemination and survival strategy [58]. Although the functions of PE_PGRS25 and PE_PGRS57 proteins are currently unknown, the observed alterations in the absence/presence of gene profiles suggest their potential involvement in driving antigenic diversity among different strains of *M. tuberculosis* associated with the PTB phenotype. This antigenic diversity contributes to the bacterial colonization of the lungs.

The hypothetical role attributed in this study to each gene associated with the PTB/EPTB clinical phenotypes suggests a primary pulmonary infection by the pathogen, followed by either the loss or acquisition, depending on the clinical phenotype, of the ability to disseminate to other parts of the body. It should be noted that EPTB can occur independently without the presence of symptoms or the development of PTB [59]. However, it is important to note that the confirmed natural route of transmission for *M. tuberculosis* is through the inhalation of aerosols containing the pathogen, which allows it to enter the host body via the respiratory tract [60,61,62]. Thus, it can be assumed that all the EPTB strains whose genomes were analyzed in this study entered the host through aerosol inhalation. Unfortunately, the metadata associated with several of the analyzed genomes do not provide sufficient information in this regard. Currently, it is not possible to establish gene–phenotype relationships that can independently distinguish every organ involved in EPTB. The unequal representation of genomes from EPTB strains associated with each potentially affected organ limits the capacity for conducting robust statistical analyses in this regard.

Lastly, it would be interesting to conduct an analysis of genes associated with EPTB to explore potential relationships with host factors, such as gender, age, and immune status (including BCG vaccination), which have been implicated as predisposition factors in the development of EPTB [60]. Currently, conducting such an analysis has not been possible due to the lack of associated metadata for most of the genomes deposited in public databases. It would be highly desirable for curated databases to include host clinical information to significantly enhance the ability to conduct robust epidemiological analyses and explore clinical associations.

## 5. Conclusions

The obtained results demonstrate significative genomic differences between PTB and EPTB strains. The PTB phenotype was found to be associated with deletions of *hspR*, *pe_pgrs5*, *pe_pgrs25*, *pe_pgrs57*, *Rv2550c*, *Rv3470c*, *Rc1759c*, and *plcD* genes. These deletions have the potential to induce changes in the processes related to the dormant stage and stress response. The EPTB phenotype was found to be associated with the presence of the *aftC* gene and the absence of the *aceaA*, *esxR*, *ppe50*, and *plcA* genes. These genetic variations can lead to changes in the fatty acid metabolism processes, conferring phenotypic advantages associated with extrapulmonary spread. The accessory genome analysis conducted in this study complements previous pangenome approaches in MTB, allowing for the detection of SNPs and rearrangements associated with the TB phenotype of *M. tuberculosis* strains. In this regard, our analysis revealed that the deletion of the genes associated with the EPTB phenotype can lead to specific metabolic profiles, generating advantages for the extrapulmonary spread of the strains. The bioinformatic analyses conducted in this work on the *M. tuberculosis* pangenome are useful for guiding the design of experimental strategies and helping to find genetic determinants and their interactions that contribute to the emergence of PTB/EPTB phenotypes.

## Figures and Tables

**Table 1:** Significant genes associated with PTB and EPTB phenotypes.

	Gene	Product	Functional Classification	*p*-Value	OR*	CI* 95%	Association
Virulence	*Rv0353*/*hspR*	Heat shock protein transcriptional repressor HspR	Virulence	0.0483	1.5	1.0034 to 2.4059	PTB
	*Rv1755c*/*plcD*	Phospholipase C	Virulence	0.0006	2	1.3487 to 2.9849	PTB
	*Rv1915*/*aceA*	Isocitrate lyase AceAa	Virulence	0.0005	2.03	1.3606 to 3.0357	EPTB
	*Rv2351c*/*plcA*	Phospholipase C	Virulence	0.007	2.78	1.3136 to 5.9075	EPTB
	*Rv3019c*/*esxR*	Secreted ESAT-6 like protein EsxR	Virulence	0.0132	1.6	1.1010 to 2.2783	EPTB
	*Rv2550c*	Antitoxin VapB20	Virulence	0.001	2.8	1.5405 to 5.5661	PTB
							
Other	*Rv3135*/*ppe50*	PPE family protein PPE50	Pe/ppe/pgrs	<0.0001	6.4	1.6896 to 4.5763	EPTB
	*Rv0297*/*pe_pgrs5*	PE-PGRS family protein PE PGRS5	Pe/ppe/pgrs	0.0303	1.5	1.0419 to 2.2758	PTB
	*Rv1396c*/*pe_pgrs25*	PE-PGRS family protein PE PGRS25	Pe/ppe/pgrs	0.0255	1.6	1.0553 to 2.2802	PTB
	*Rv1759c*/*wag22*	PE-PGRS family protein Wag22	Pe/ppe/pgrs	<0.0001	2.4	1.6427 to 3.4220	PTB
	*Rv3514*/*pe_pgrs57*	PE-PGRS family protein PE PGRS57	Pe/ppe/pgrs	0.0201	1.5	1.0679 to 2.1640	PTB
	*Rv3740c*	Triacylglycerol synthase (diacylglycerol acyltransferase)	Lipid metabolism	0.0266	1.5	1.0516 to 2.2586	PTB

OR*, odds ratio; CI*, confidence interval; EPTB, extrapulmonary tuberculosis; PTB, pulmonary tuberculosis.

## Data Availability

The whole-genome shotgun project of MYC strains has been deposited in NCBI SRA under the Bioproject accession number PRJNA880281. Public datasets used in this work are deposited under the SRA accession numbers cited in Table S1.

## Supplementary Materials

The following supporting information can be downloaded at: https://www.mdpi.com/article/10.3390/microorganisms11061495/s1. Table S1: Statistics of the *Mycobacterium tuberculosis* assembled genomes, Table S2: Pyseer analysis of gene–phenotype associations, Table S3: Sublineage classification of *Mycobacterium tuberculosis* PTB clades, Table S4: Sublineage classification of *Mycobacterium tuberculosis* EPTB clades.
