# Supplementary material for: Pangenome Reconstruction of Mycobacterium tuberculosis as a Guide to Reveal Genomic Features Associated with Strain Clinical Phenotype
Source: Microorganisms. 2023 Jun 4;11(6):1495. doi: 10.3390/microorganisms11061495 (PMC10300688; doi:10.3390/microorganisms11061495)
Supplement: Supplementary file 1 [file microorganisms-11-01495-s001.zip › microorganisms-2299267-supplementary.pdf]

Table S1. Statistics of the *Mycobacterium tuberculosis* assembled genomes.

Sample ID	Number of contigs	Total length	GC (%)	Genome fraction (%)	# N's per 100 kbp
ERR1144992	101	4523082	65,57	97,881	4035,79
ERR1144993	5	4516751	65,57	97,834	4009,54
ERR1144994	135	4517864	65,55	97,685	4131,47
ERR1144995	118	4512367	65,56	97,897	3941,97
ERR1950087	165	4484893	65,51	97,227	4089,02
ERR2229064	353	4589631	65,54	93,761	3607,81
ERR2229066	347	4550270	65,6	93,493	3070,41
ERR2229322	195	4683593	65,56	86,754	3706,96
ERR2229324	213	4900776	65,55	83,112	6075,65
ERR2229359	350	4522314	65,56	92,347	3511,92
ERR2229361	398	4587225	65,54	90,785	4973,46
ERR2229363	165	4664662	65,48	90,095	2988,6
ERR2229365	401	4662682	65,54	90,757	4174,1
ERR2229366	106	4724837	65,52	89,544	3626,33
ERR2229367	229	4569020	65,53	92,656	3297,95
ERR2229374	354	4775794	65,56	89,195	3287,33
ERR2229774	277	4739785	65,55	91,332	3262,66
ERR2229808	143	4471989	65,47	97,38	3556,99
MYC36	120	4501126	65,54	92,375	8792,64
MYC52	125	4473036	65,54	96,881	3914,05
MYC61	126	4504014	65,56	91,119	10257,94
MYC72	126	4341393	65,54	95,509	2255,17
MYC75	133	4519365	65,52	97,368	4472,6
MYC78	127	4468615	65,54	91,658	9084,76
MYC87	112	4499176	65,53	92,303	8806,3
MYC93	122	4486405	65,54	96,793	4085,74
MYC96	123	4529861	65,53	97,332	4404,82
MYC136	114	4499957	65,52	97,359	3854,7
SRR1002679	216	4458568	65,58	97,348	2952,67
SRR1002684	139	4603235	65,46	94,232	7299,65
SRR1002686	148	4483746	65,58	97,802	2793,11
SRR1002689	200	4441959	65,57	97,939	1856,75
SRR1002690	183	4503558	65,55	97,527	3477,61
SRR1510036	139	4550604	65,51	97,67	4427,39
SRR1510049	133	4544195	65,48	97,594	4573,97
SRR1510057	133	4519015	65,5	97,505	3960,55
SRR1510058	192	4565515	65,52	94,834	3593,72
SRR1510060	126	4549385	65,52	97,355	4805,09
SRR1510062	134	4496925	65,53	97,789	3339,24
SRR1510071	139	4476503	65,49	97,643	2917,72
SRR1573725	137	4490225	65,44	96,196	4934,72
SRR1573727	100	4583317	65,52	97,171	5564,27

SRR1573728	197	4492545	65,46	97,289	3860,91
SRR1573729	100	4593165	65,52	97,835	5118,85
SRR1573730	108	4609634	65,52	97,624	5999,54
SRR1573733	121	4520500	65,44	97,243	3810,6
SRR5125074	87	4515676	65,59	97,914	3841,86
SRR5125075	103	4437787	65,59	98,164	1863,09
SRR5125076	89	4515851	65,59	97,945	3806,72
SRR5125077	127	4513866	65,59	97,923	3819,59
SRR5125078	86	4515286	65,59	97,866	3883,96
SRR5341272	83	4526455	65,56	91,469	10056,32
SRR5341273	91	4526852	65,58	98,137	3456,53
SRR5341274	86	4571653	65,54	89,971	12312,72
SRR5341275	102	4545602	65,54	97,639	4492,41
SRR5341276	107	4525417	65,59	98,289	3464,83
SRR5341277	104	4437570	65,56	97,5	2426,42
SRR5709738	77	4552605	65,59	97,559	4611,27
SRR5709739	81	4429023	65,6	100	1747,88
SRR5709740	56	4518700	65,56	83,873	17578
SRR5709741	79	4430455	65,59	97,93	1805,62
SRR5709742	80	4432398	65,59	97,79	1988,54
SRR5709744	83	4528365	65,59	98,357	3245,76
SRR5709745	79	4526103	65,58	97,986	3614,88
SRR5709746	80	4431924	65,59	97,941	1799,38
SRR5709747	70	4526797	65,56	95,152	6418,64
SRR5709749	78	4431390	65,6	97,829	1796,91
SRR5709753	79	4431454	65,59	97,942	1801,71
SRR5709759	71	4419744	65,59	97,621	2041,77
SRR5709761	86	4469438	65,59	97,001	3553,36
SRR5709763	155	4516310	65,37	96,453	4976,65
SRR5709770	87	4431285	65,59	97,626	1974,39
SRR5709773	79	4430735	65,59	97,858	1779,77
SRR5709776	76	4431650	65,58	90,411	9324,29
SRR5709777	83	4561405	65,56	100	7863,19
SRR5709778	75	4429997	65,6	97,88	1848,47
SRR5709779	79	4522558	65,59	98,228	3376,58
SRR5709783	81	4430671	65,59	97,904	1796,7
SRR5709784	74	4431077	65,59	97,853	1774,47
SRR5709785	73	4431376	65,59	97,986	1758,87
SRR5709786	79	4431027	65,59	98,018	1765,62
SRR5709787	79	4431575	65,59	97,925	1863,6
SRR5709788	76	4430614	65,59	97,957	1718,81
SRR5709789	75	4568967	65,57	96,486	5930,29
SRR5709790	74	4430168	65,59	97,838	1838,55
SRR5709791	73	4527213	65,59	98,014	3839,78
SRR5709792	71	4572028	65,56	96,906	5596,23
SRR5709793	106	4441596	65,57	97,709	2094,54

SRR5709794	81	4428071	65,59	97,823	1695,25
SRR5709796	89	4423429	65,62	100	3263,92
SRR5709798	85	4505794	65,59	96,381	4980,92
SRR5709799	74	4431885	65,61	97,922	1805,78
SRR5709800	79	4524731	65,59	98,316	3146,44
SRR5709801	78	4427879	65,59	90,546	9145,1
SRR5709802	75	4430589	65,6	97,964	1679,46
SRR5709804	75	4430330	65,59	97,813	1851,08
SRR5709805	75	4429384	65,59	97,912	1754,56
SRR5709806	77	4428770	65,6	97,886	1750,78
SRR5709812	85	4431446	65,6	97,852	1861,42
SRR5709813	75	4431165	65,6	97,864	1847,69
SRR5709820	78	4431080	65,59	97,851	1808,18
SRR5709823	164	4430718	65,6	97,813	1876,08
SRR5709828	76	4431643	65,6	97,899	1873,59
SRR5709829	77	4430831	65,59	98,005	1738,93
SRR5709830	92	4433793	65,59	97,887	1825,66
SRR5709831	80	4430749	65,59	97,775	1884,58
SRR5709832	70	4526398	65,58	97,983	3567,38
SRR5709833	89	4527323	65,59	98,412	3240,88
SRR5709834	69	4520894	65,57	95,004	6391,52
SRR5709835	88	4513224	65,61	89,22	12236,82
SRR5709836	74	4425219	65,6	97,802	1674,43
SRR5709837	95	4439247	65,58	97,775	2199,83
SRR5709838	102	4568809	65,51	100	5471,82
SRR5709850	79	4431336	65,59	97,787	1911,93
SRR5709851	88	4431058	65,58	97,799	1926,83
SRR5709852	78	4430205	65,6	97,982	1745,04
SRR5709853	81	4429554	65,59	97,803	1824,68
SRR5709854	72	4523085	65,58	95,527	5923,57
SRR5709855	84	4473518	65,58	97,884	2791,4
SRR5709856	94	4670975	65,57	97,875	6617,72
SRR5709857	84	4434335	65,58	95,333	4275,64
SRR5709858	78	4522239	65,58	95,186	6297,26
SRR5709859	69	4431005	65,59	90,606	9067,36
SRR5709860	87	4424099	65,62	96,338	3333,29
SRR5709861	90	4554111	65,57	97,318	4979,39
SRR5709862	74	4430541	65,6	97,984	1769,63
SRR5709864	98	4451426	65,56	97,706	2460,94
SRR5709875	83	4432697	65,59	98,03	1751,3
SRR5709877	77	4427993	65,6	97,854	1800,95
SRR5709881	80	4431943	65,59	97,73	1971,8
SRR5709884	166	4442506	65,37	92,22	7495,32
SRR5709885	161	4466939	65,38	95,918	4540,36
SRR5709888	154	4461630	65,36	96,523	3756,56
SRR5709889	185	4449535	65,34	95,797	4169,67

SRR5709892	162	4488304	65,36	96,299	4507,32
SRR5709896	75	4523353	65,57	95,043	6335,59
SRR5709903	79	4432245	65,59	97,824	1880,63
SRR5709907	76	4429587	65,6	97,977	1784,86
SRR5709910	81	4422102	65,62	96,521	3149,59
SRR5709911	78	4431655	65,59	97,903	1865,22
SRR5709912	79	4422381	65,62	96,47	3171,71
SRR5709921	74	4431015	65,6	97,853	1835
SRR5709922	94	4437468	65,57	97,77	2105,62
SRR5709927	83	4431227	65,6	97,851	1699,87
SRR5709928	163	4468535	65,36	96,194	4296,64
SRR5709929	79	4422104	65,62	96,421	3238,14
SRR5709931	172	4453410	65,35	96,308	3740,17
SRR5709933	154	4482730	65,39	95,395	5421,23
SRR5709936	74	4431930	65,6	97,875	1813,52
SRR5709938	130	4508588	65,4	87,565	14028,32
SRR5709939	91	4492666	65,58	97,699	3293,83
SRR5709940	85	4441268	65,58	96,429	3536,08
SRR5709943	74	4429955	65,59	97,861	1790,33
SRR5709946	73	4528493	65,58	98,038	3563,75
SRR5709948	91	4431984	65,6	97,906	1862,66
SRR5709952	74	4428149	65,6	97,876	1716,5
SRR5709953	78	4431911	65,59	100	1777,16
SRR5709954	80	4431417	65,59	97,884	1834,83
SRR5709956	76	4432783	65,59	97,994	1851,86
SRR5709959	78	4432214	65,59	97,863	1946,16
SRR5709960	155	4440229	65,37	96,513	3376,13
SRR5709961	79	4462091	65,59	97,028	3351,48
SRR5709962	75	4429892	65,59	97,923	1797,83
SRR5709964	85	4430801	65,59	97,867	1751,31
SRR5709967	207	4491741	65,57	97,748	3199,05
SRR5709971	65	4517787	65,56	83,898	17499,81
SRR5709972	70	4513678	65,59	95,541	6148,84
SRR5709973	62	4508855	65,57	95,435	6395,75
SRR5709974	78	4431258	65,59	97,764	1922,19
SRR5709975	74	4523546	65,59	98,408	3152,24
SRR5709976	81	4433160	65,58	97,915	1829,08
SRR5709977	83	4432353	65,59	97,885	1784,54
SRR5709978	71	4553529	65,58	97,63	4548,69
SRR5709979	71	4551318	65,54	93,206	8872,02
SRR5709980	72	4433478	65,59	97,88	1889,87
SRR5709983	75	4436239	65,57	97,48	2344,55
SRR5709984	64	4519960	65,55	88,236	13057,66
SRR5709985	81	4429186	65,6	98,037	1726,05
SRR5709986	78	4430806	65,58	97,896	1757,4
SRR5709991	74	4420726	65,59	97,753	1823,34

SRR5709995	86	4523525	65,56	91,203	10266,26
SRR5709997	87	4523293	65,55	88,678	12670,97
SRR5709998	71	4569536	65,56	96,595	5764,37
SRR5709999	68	4518563	65,56	88,288	13017,21
SRR5710000	70	4431049	65,59	90,707	9014,8
SRR5710001	67	4524451	65,59	98,08	3463,56
SRR5710002	81	4524611	65,58	98,159	3485,45
SRR5710003	69	4518032	65,57	91,028	10232,93
SRR5710004	74	4523704	65,58	97,865	3634,92
SRR5710005	69	4525087	65,59	97,963	3642,78
SRR5710006	85	4432220	65,59	97,84	1894,2
SRR5710009	83	4429854	65,59	97,907	1750,71
SRR5710011	77	4430612	65,6	97,868	1756,71
SRR5710012	77	4424488	65,61	96,407	3217,25
SRR5710016	80	4429262	65,59	97,814	1879,48
SRR5710026	143	4508806	65,39	95,987	5212,05
SRR5710028	151	4487636	65,4	96,542	4116,58
SRR5710029	71	4429688	65,58	90,722	9024,18
SRR5710030	84	4427899	65,59	97,673	1982,54
SRR5817466	107	4472453	65,55	97,962	2654,34
SRR5817467	131	4506472	65,54	97,947	3457,69
SRR6256975	154	4505385	65,47	97,122	4062,18
SRR6256976	145	4527912	65,51	91,134	10357,33
SRR6256978	113	4496339	65,55	94,937	3159,15
SRR6256980	79	4517695	65,61	91,743	9652,2
SRR6256983	171	4458628	65,47	96,76	3436,69
SRR6256984	137	4501572	65,48	95,981	5035,37
SRR6256985	130	4441409	65,57	97,183	3140,13
SRR6256986	110	4422952	65,6	97,582	2411,74
SRR6256989	122	4422154	65,6	97,905	2071,16
SRR6256990	88	4485557	65,6	98,305	3192
SRR6256992	194	4430441	65,57	97,347	2739,75
SRR6256993	83	4452469	65,6	97,115	3569,26
SRR6257006	104	4467972	65,59	97,862	2580,5
SRR6257007	99	4421314	65,6	97,935	1982,92
SRR6257013	96	4418526	65,6	97,817	2089,68
SRR6257018	87	4418067	65,6	100	2109,61
SRR6257024	117	4467869	65,6	97,887	2525,34
SRR6257025	86	4457596	65,6	98,039	2690,01
SRR6257027	97	4420889	65,6	98,015	1910,93
SRR6257028	72	4442582	65,58	97,65	2943
SRR6257030	100	4435926	65,59	97,991	1719,8
SRR6257031	122	4422936	65,59	97,592	2361,58
SRR6257032	107	4436623	65,6	97,908	2345,64
SRR6257034	90	4445961	65,59	97,198	3284,42
SRR6257056	92	4433035	65,59	97,93	1737,52

SRR6257080	95	4445916	65,59	98,01	11078,88
SRR6257081	107	4451774	65,6	97,465	3177,54
SRR6257082	110	4462752	65,59	97,868	2984,64
SRR6257084	129	4424159	65,6	97,075	2840,95
SRR6257085	99	4420998	65,6	97,997	1968,94
SRR6257086	91	4525211	65,57	93,703	8507,36
SRR6257087	126	4449852	65,59	97,741	2723,53
SRR6257091	92	4434840	65,6	98,073	1660,38
SRR6257097	276	4434600	65,6	98,055	1690,8
SRR6257100	124	4422785	65,59	97,916	2030,39
SRR6257102	116	4441267	65,6	97,856	2580,77
SRR6257105	187	4445029	65,47	96,189	4319,7
SRR6257106	101	4421434	65,59	97,704	2193,97
SRR6257107	166	4452319	65,6	96,866	3779,13
SRR6257109	110	4422505	65,59	97,782	2115,66
SRR6339646	128	4432571	65,53	97,423	2398,07
SRR6367399	113	4544599	65,52	97,531	4339,37
SRR6397999	95	4525455	65,6	98,209	3489,86
SRR6480314	122	4520097	65,43	96,704	5009,38
SRR6480315	110	4523279	65,43	95,227	6665,14
SRR6480316	123	4469796	65,46	96,738	3875,61
SRR6480318	131	4438102	65,42	93,569	6731,39
SRR6480321	75	4563137	65,6	98,043	4975,61
SRR6480322	91	4434737	65,58	97,399	2456,74
SRR6480326	86	4440354	65,57	97,758	2239,8
SRR6480327	91	4482838	65,6	95,458	5555,79
SRR6480328	85	4516048	65,58	97,068	4504,03
SRR6480329	87	4454165	65,58	97,794	2360,13
SRR6480331	103	4528006	65,58	97,368	1897,5
SRR6480333	133	4434062	65,46	96,83	3083,79
SRR6480336	121	4465869	65,41	96,277	4588,92
SRR6480339	96	4566556	65,48	96,201	6743,09
SRR6480342	119	4592706	65,37	95,604	7889,88
SRR6480343	153	4514909	65,34	96,409	5577,52
SRR6480344	139	4489552	65,37	96,557	4712,07
SRR6480345	129	4529597	65,37	95,56	6673,42
SRR6480354	114	4570153	65,45	95,257	6724,96
SRR6480357	128	4501410	65,39	96,636	5021,4
SRR6480358	115	4493984	65,44	95,074	6039,54
SRR6480359	139	4505292	65,38	95,347	6014,82
SRR6480362	130	4557153	65,42	96,024	6720,29
SRR6480363	109	4563011	65,49	97,183	5823,9
SRR6480364	110	4472695	65,49	97,075	3635,37
SRR6480365	112	4542322	65,47	96,974	5221,21
SRR6480366	115	4435051	65,5	97,304	2592,55
SRR6480367	194	4463115	65,47	97,219	3305,14

SRR6480369	122	4436009	65,48	97,305	2599,88
SRR6480370	97	4511388	65,49	97,596	4320,42
SRR6480371	117	4550530	65,47	97,516	5025,33
SRR6480372	118	4429934	65,48	97,193	2623,65
SRR6480373	106	4473461	65,54	95,42	5416,21
SRR6480376	117	4620243	65,45	94,387	9481,06
SRR6480377	130	4472830	65,42	96,21	4509,16
SRR6480378	119	4524387	65,5	95,85	6074,72
SRR6480381	124	4457678	65,53	97,251	3166,65
SRR6480382	128	4468694	65,38	95,117	5554,69
SRR6480385	95	4542385	65,55	98,156	4068,74
SRR6480389	127	4568006	65,36	92,117	10853,6
SRR6480390	116	4535151	65,35	94,488	7541,6
SRR6480391	134	4525869	65,37	96,099	6125,56
SRR6480394	142	4486624	65,36	96,068	4972,76
SRR6480395	145	4527775	65,36	96,612	5400,53
SRR6480396	125	4566937	65,36	94,046	8804,46
SRR6480397	128	4497749	65,48	97,275	3973,14
SRR6480398	141	4541919	65,38	96,559	5922,12
SRR6480399	124	4540496	65,43	94,075	8358,07
SRR6480401	130	4478011	65,44	96,766	4057,49
SRR6480402	99	4549349	65,44	91,461	11078,88
SRR6480403	122	4534250	65,44	97,132	5278,67
SRR6480404	136	4553943	65,37	94,662	7829,39
SRR6480405	135	4486185	65,38	96,502	4557,95
SRR6480406	280	4465625	65,46	96,277	4037,76
SRR6480407	139	4542124	65,38	96,296	5908,8
SRR6480408	125	4449222	65,47	97,072	3201,19
SRR6480411	130	4596702	65,41	96,567	7089,5
SRR6480412	124	4471707	65,37	95,268	5499,18
SRR6480414	132	4444229	65,41	96,442	3599,59
SRR6480415	113	4572900	65,43	96,774	6440,57
SRR6480416	134	4506365	65,4	93,173	8163,39
SRR6480418	130	4512843	65,35	93,911	7651,43
SRR6480420	121	4453631	65,42	96,437	3844,86
SRR6480421	129	4509050	65,43	96,477	5000,45
SRR6480424	139	4500173	65,37	96,279	4972,88
SRR6480427	127	4659137	65,38	94,844	10031,21
SRR6480428	118	4529623	65,41	96,766	5219,82
SRR6480430	131	4487196	65,37	96,614	4610,07
SRR6480431	119	4532686	65,42	96,554	5653,42
SRR6480432	146	4468051	65,4	96,436	4254,88
SRR6480433	127	4559519	65,36	94,415	8172,05
SRR6480434	131	4483609	65,38	96,519	4414,41
SRR6480435	147	4538219	65,38	95,443	6997,77
SRR6480436	127	4615879	65,37	96,586	7274,71

SRR6480437	129	4517998	65,38	95,567	6462,55
SRR6480438	137	4498164	65,41	97,028	4244,75
SRR6480439	134	4519974	65,37	95,945	5918,91
SRR6480440	117	4533642	65,47	96,297	6026,46
SRR6480441	117	4611572	65,46	95,854	8086,05
SRR6480444	116	4557130	65,45	97,328	5314,42
SRR6480445	116	4521048	65,45	95,435	6383,7
SRR6480446	104	4541046	65,45	95,967	6558,07
SRR6480447	119	4564261	65,4	96,469	6380,07
SRR6480448	117	4540421	65,46	95,614	6654,32
SRR6480450	105	4557334	65,4	95,416	7448,72
SRR6480452	111	4535918	65,49	97,321	5173,99
SRR6480453	126	4489268	65,42	96,338	4700,03
SRR6480454	131	4581360	65,38	95,415	7914,7
SRR6480455	127	4509322	65,39	95,261	6446,36
SRR6480456	122	4574021	65,39	95,219	7924,32
SRR6480457	123	4555656	65,39	96,882	5892,74
SRR6480458	119	4641859	65,39	95,067	9100,77
SRR6480459	119	4499153	65,41	95,44	5816,74
SRR6480460	133	4486433	65,36	94,99	6370,74
SRR6480462	110	4456344	65,34	98,08	5162,64
SRR6480463	118	4551144	65,45	97,501	5162,64
SRR6480466	134	4587501	65,39	93,884	9501,38
SRR6480468	136	4615972	65,38	96,835	7169,74
SRR6480469	137	4520637	65,37	95,67	6379,74
SRR6480471	138	4525496	65,38	96,737	5240,69
SRR6480472	133	4556628	65,38	95,905	6860,29
SRR6480473	142	4462228	65,39	96,653	3991,3
SRR6480475	130	4670038	65,37	95,705	9200,67
SRR6480476	139	4479342	65,41	94,713	6093,55
SRR6480477	159	4455229	65,35	96,064	4261,06
SRR6480478	116	4485868	65,39	96,533	4767,86
SRR6480479	138	4479134	65,38	95,014	5847,05
SRR6480480	143	4515147	65,37	96,844	5166,74
SRR6480481	146	4510212	65,35	96,587	5277,53
SRR6480482	143	4534387	65,37	96,286	5917,01
SRR6480483	121	4433648	65,49	97,081	2839,29
SRR6480484	121	4436077	65,5	97,267	2587,13
SRR6480485	110	4552628	65,46	97,504	4979,59
SRR6480486	104	4611732	65,5	94,834	8835,27
SRR6480487	119	4432527	65,48	97,249	2604,2
SRR6480488	117	4453020	65,48	97,121	3193,07
SRR6480489	117	4523049	65,49	94,418	7525,26
SRR6480490	103	4538293	65,49	97,183	5178,47
SRR6480491	109	4539267	65,48	97,661	4803,88
SRR6480492	113	4455704	65,48	97,187	3138,99

SRR6480494	157	4524503	65,36	96,072	5833,04
SRR6480495	119	4582649	65,45	96,974	6376,9
SRR6480497	124	4491030	65,45	96,798	4295,36
SRR6480498	126	4589255	65,38	96,32	7123,38
SRR6480499	119	4571506	65,41	95,478	7454,24
SRR6480500	120	4527522	65,38	95,578	6579,65
SRR6480501	138	4527740	65,37	95,496	6352,29
SRR6480502	134	4531604	65,37	94,718	7508,53
SRR6480507	131	4490913	65,38	94,838	6245,48
SRR6480508	119	4578806	65,38	94,884	8241,28
SRR6480509	125	4504599	65,45	97,055	4376,31
SRR6480510	127	4441605	65,44	96,492	3616,26
SRR6480512	125	4528687	65,41	95,768	6344,6
SRR6480513	132	4549473	65,43	97,44	4711,47
SRR6480516	121	4525400	65,44	96,899	5333,45
SRR6480518	125	4460960	65,45	97,027	3426,57
SRR6480520	124	4553511	65,38	95,308	7410,39
SRR6480521	151	4450106	65,37	96,415	3883,91
SRR6480522	152	4449450	65,35	96,239	4038,79
SRR6480525	136	4580276	65,36	96,692	6117,95
SRR6480526	133	4523566	65,37	96,357	5485,3
SRR6480527	141	4457382	65,37	96,428	3971,95
SRR6480528	136	4577318	65,37	95,711	7559,03
SRR6480529	109	4529162	65,51	96,349	5930,04
SRR6480530	105	4527131	65,53	96,328	5824,79
SRR6480531	104	4514312	65,52	98,123	3739,79
SRR6480533	124	4439421	65,52	97,435	2542,63
SRR6480534	119	4449969	65,5	96,453	3727,19
SRR6480535	99	4551304	65,5	97,667	4908,62
SRR6480537	133	4524432	65,39	96,844	5279,87
SRR6480539	131	4574886	65,39	94,809	8234,72
SRR6480540	125	4436267	65,46	97,076	2844,19
SRR6480541	127	4441361	65,53	97,449	2539,11
SRR6480542	120	4530383	65,46	97,303	4866,76
SRR6480543	136	4460726	65,38	96,315	4191,27
SRR6480544	118	4517100	65,43	97,317	4491,4
SRR6480545	130	4467703	65,51	96,308	4282,83
SRR6480546	109	4512285	65,49	97,659	4047,2
SRR6480548	117	4521081	65,48	97,757	4099,75
SRR6480549	110	4478700	65,49	95,89	4871,21
SRR6480550	117	4451468	65,49	97,305	2948,98
SRR6480552	110	4588909	65,5	95,363	7717,06
SRR6480555	90	4465032	65,57	97,005	3482,44
SRR6480557	80	4518720	65,54	91,326	10326,18
SRR6480562	80	4563366	65,59	98,425	4264,22
SRR6480563	81	4563215	65,58	97,264	5704,68

SRR6480565	126	4438374	65,53	97,5	2510,85
SRR6480566	115	4431680	65,51	96,269	3566,77
SRR6480567	100	4530527	65,52	97,451	4743,82
SRR6480568	111	4565652	65,5	96,957	6072,74
SRR6480573	101	4467012	65,56	94,861	6185,43
SRR6480574	122	4436212	65,5	97,225	2697,12
SRR6480575	109	4526136	65,53	98,087	3961,75
SRR6480576	109	4479553	65,52	97,344	3571,2
SRR6480582	111	4548582	65,42	96,865	5479,53
SRR6480583	126	4431428	65,49	97,26	2606,02
SRR6480584	127	4448674	65,48	96,928	3206,57
SRR6480585	115	4519373	65,45	94,293	7499,78
SRR6480595	90	4563775	65,58	97,757	5225,5
SRR6480596	95	4671233	65,35	97,85	6002,7
SRR6480601	124	4609863	65,4	92,502	10928,26
SRR6480602	134	4514765	65,43	95,844	5762,51
SRR6480603	129	4511835	65,42	95,891	6002,7
SRR6480604	126	4558732	65,43	93,633	9199,14
SRR6480605	152	4467153	65,37	96,844	4159,05
SRR6480606	120	4432196	65,53	97,045	2791,98
SRR6480607	120	4565445	65,43	95,38	7657,7
SRR6480608	119	4564857	65,53	97,647	5412,83
SRR6480609	116	4486965	65,47	97,556	3549,77
SRR6480610	81	4520633	65,58	98,2	3943,81
SRR6480611	88	4513888	65,58	97,06	4575,5
SRR6480612	133	4431258	65,5	97,221	2651,26
SRR6480617	120	4566719	65,46	97,352	5740,51
SRR6480619	122	4515576	65,47	94,551	7160,44
SRR6480620	118	4524185	65,52	97,591	4620,99
SRR6480621	81	4563239	65,59	97,337	5598,57
SRR6480622	91	4529566	65,55	97,794	3900,51
SRR6480623	110	4434946	65,51	97,038	3081,73
SRR6480624	125	4468507	65,51	97,366	3249,94
SRR6480626	102	4609674	65,52	94,391	9472,75
SRR6480627	108	4501295	65,5	97,03	4475,78
SRR6480628	126	4551590	65,35	95,592	7076,91
SRR6480629	120	4483292	65,51	97,293	3571,64
SRR6480631	121	4484986	65,49	96,321	4707,75
SRR6480632	129	4460095	65,49	97,249	3256,19
SRR6480634	120	4523435	65,42	97,075	5042,85
SRR6914119	68	4431567	65,6	97,983	1562,99
SRR6914120	74	4432784	65,61	97,959	1749,53
SRR6914121	70	4431612	65,6	98,151	1528,63
SRR6914122	69	4439579	65,59	98,09	1654,39
SRR6914123	68	4532007	65,58	91,792	9929,79
SRR8327216	88	4427482	65,59	97,727	1955,83

SRR8330159	86	4428143	65,6	97,764	2006,42
SRR8335024	95	4428365	65,59	97,738	2009,36
SRR8335313	83	4427703	65,6	97,804	1962,76
SRR8344409	94	4429388	65,59	97,697	2066,7
SRR8345980	86	4427342	65,59	97,758	1941,3
SRR8346061	287	4474679	65,57	97,241	3533,39
SRR8346382	94	4429145	65,59	97,596	2136,08
SRR8347539	91	4428412	65,6	97,747	2025,11
SRR8348431	88	4429248	65,59	97,646	2011,74
SRR8348943	85	4429271	65,6	97,822	2000,08
SRR8351921	76	4430105	65,59	97,805	1947,99
SRR8351971	86	4428643	65,6	97,604	2164,46
SRR8352208	87	4428017	65,59	97,752	1945,18
SRR8353274	103	4433387	65,59	97,72	2132,43
SRR8353479	90	4427555	65,59	97,711	2022,52
SRR8354461	92	4428643	65,6	97,69	2083,1
SRR8354716	90	4428028	65,6	97,619	2039,89
SRR8357275	83	4430065	65,59	97,821	1980,58
SRR8358462	90	4427688	65,59	97,803	1990,63
SRR8358501	209	4428644	65,59	97,584	2107,78
SRR8366172	102	4429719	65,59	97,662	2170,45
SRR8366603	96	4441239	65,6	97,598	2389,02
SRR8369849	89	4427436	65,59	97,77	1947,06
SRR8369893	89	4428337	65,6	97,695	2089,5
SRR8370129	88	4427497	65,59	97,714	1957,54
SRR8375802	99	4429059	65,59	97,656	2139,21
SRR8427096	83	4428189	65,59	97,85	1941,31
SRR8433589	94	4428736	65,6	97,635	2146,93
SRR8434639	97	4429027	65,59	97,501	2160,7
SRR8434867	84	4427985	65,6	97,745	1966,45
SRR8439237	87	4430495	65,59	97,722	2010,16
SRR8439315	137	4430613	65,59	97,632	2148,15
SRR8662666	112	4495923	65,53	97,664	3407,62
SRR8662668	106	4460455	65,52	92,988	7227,45
SRR974700	84	4434642	65,6	97,945	1812,46
SRR993002	74	4324609	68,95	8,62	90977,77
SRR993140	83	4517940	65,61	91,738	9658,21
SRR998140	174	4574961	65,52	97,488	5173,71
SRR998855	364	4507722	65,52	95,307	5483,24
SRR998856	154	4505385	65,47	97,122	4062,18
SRR998857	145	4527912	65,51	91,128	10357,36
SRR998858	197	4518952	65,52	97,322	3998,07
SRR998860	171	4458628	65,47	96,76	3436,69
SRR998861	137	4501572	65,48	95,981	5035,37

Table S2. Pyseer analysis of gene-phenotype associations.

EPTB phenotype

variant	af*	filter-pvalue**	lrt-pvalue***	beta****
aftC	8.99E-01	3.86E-09	2.95E-10	2.51E+00
aceAa	2.73E-01	1.47E-03	3.16E-04	-8.15E-01
plcA	9.34E-01	2.91E-02	2.13E-02	-5.28E-01
esxR	6.18E-01	1.69E-02	8.01E-04	-6.53E-01
PPE50	7.91E-01	4.38E-14	8.79E-14	-1.95E+00

PTB phenotype

variant	af*	filter-pvalue**	lrt-pvalue***	beta****
hspR	7.86E-01	1.40E-02	2.76E-02	5.26E-01
plcC	9.40E-01	3.62E-02	2.08E-02	-5.67E-01
vapB20	1.64E-01	1.17E-05	4.08E-03	8.56E-01
PE_PGRS5	7.08E-01	2.62E-02	1.48E-05	1.57E+00
PE_PGRS25	6.96E-01	2.25E-01	2.24E-02	7.96E-01
Rv1759c	5.93E-01	9.70E-01	8.39E-01	-3.82E-02
PE_PGRS57	5.20E-01	4.15E-02	3.87E-03	7.26E-01
Rv3740c	3.20E-01	2.32E-01	2.45E-01	-2.47E-01

af*	Allele frequency. The proportion of samples the variant is present in.
filter-pvalue**	Association of the variant with the phenotype, unadjusted for population structure.
lrt-pvalue***	p-value of association, adjusted for population structure. This corresponds to the LRT p-value of seer.
beta****	Effect size/slope of the variant.

Table S3. Sublineage classification of *Mycobacterium tuberculosis* PTB clades.

Clade 1 EPTB	Sublineage (MTBseq)	Clade 2	Sublineage (MTBseq)
MYC75 PTB	4.3.4.2	SRR6480428 PTB	4.5
SRR1002686 PTB	4.3.3	SRR6480458 PTB	4.5
SRR5709938 PTB	4.3.3	SRR6480418 PTB	4.5
SRR6480318 PTB	4.3.3	SRR6480485 EPTB	4.5
SRR6480321 PTB	4.3.1	SRR6480390 PTB	4.5
SRR6480336 PTB	4.3.3	SRR6480582 EPTB	4.5
SRR6480343 PTB	4.3.3	SRR6480548 PTB	4.5
SRR6480345 PTB	4.3.1	SRR6480535 PTB	4.5
SRR6480357 PTB	4.3.1	SRR6480444 PTB	4.5
SRR6480362 PTB	4.3.3	SRR6480546 PTB	4.5
SRR6480389 PTB	4.3.1	SRR6480438 PTB	4.5
SRR6480391 PTB	4.3.1	SRR6480412 PTB	4.5
SRR6480411 PTB	4.3.1		
SRR6480415 PTB	4.3.1		
SRR6480427 PTB	4.3.3		
SRR6480441 PTB	4.3.1		
SRR6480494 PTB	4.3.1		
SRR6480495 PTB	4.3.1		
SRR6480529 PTB	4.3.3		
SRR6480530 PTB	4.3.1		
SRR6480539 PTB	4.3.3		
SRR6480563 PTB	4.3.1		
SRR6480568 PTB	4.3.1		
SRR6480573 PTB	4.3.4.2		
SRR6480603 PTB	4.3.1		
SRR6480604 PTB	4.3.4.2		
SRR6480607 PTB	4.3.1		
SRR6480610 PTB	4.3.3		
SRR6480617 PTB	4.3.1		
SRR6480621 PTB	4.3.1		
SRR6480634 PTB	4.3.3		
SRR6480363 PTB	4.3.1		
SRR6480385 PTB	4.3.1		
SRR6480435 PTB	4.3.3		
SRR6480437 PTB	4.3.4.2		
SRR6480456 PTB	4.3.1		
SRR6480466 PTB	4.3.1		
SRR6480468 PTB	4.3.2		
SRR6480469 PTB	4.3.1		
SRR6480472 PTB	4.3.2		
SRR6480481 PTB	4.3.2		
SRR6480528 PTB	4.3.1		
SRR6480537 PTB	4.3.3		

Table S4. Sublineage classification of *Mycobacterium tuberculosis* EPTB clades.

Clade 1 EPTB	Sublineage (MTBseq)	Clade 2	Sublineage (MTBseq)
SRR1510036 EPTB	1.2.1	MYC136 EPTB	4.1.1
SRR1510049 EPTB	1.2.1	MYC52 EPTB	4.1.1
SRR1510057 EPTB	1.2.1	MYC78 EPTB	4.1.1
SRR1510058 EPTB	1.2.1	MYC87 EPTB	4.1.1
SRR1510060 EPTB	1.2.1	SRR6256990 EPTB	4.1.2.1
SRR1510062 EPTB	1.2.1	SRR6257081 EPTB	4.1.2
SRR1510071 EPTB	1.2.1	SRR6480371 EPTB	4.1.2
SRR5709738 EPTB	1.2.1	SRR6480376 EPTB	4.1.2.1
SRR5709744 EPTB	1.2.1	SRR6480378 EPTB	4.1.2.1
SRR5709745 EPTB	1.1.1	SRR6480395 EPTB	4.1.2.1
SRR5709747 EPTB	1.2.1	SRR6480404 EPTB	4.1.2.1
SRR5709779 EPTB	1.2.1	SRR6480406 EPTB	4.8
SRR5709789 EPTB	1.1.1	SRR6480433 EPTB	4.1.2.1
SRR5709800 EPTB	1.2.1	SRR6480439 EPTB	4.1.2.1
SRR5709832 EPTB	1.1.1	SRR6480445 EPTB	4.1.2.1
SRR5709833 EPTB	1.2.1	SRR6480448 EPTB	4.1.2.1
SRR5709834 EPTB	1.1.1	SRR6480482 EPTB	4.1.2
SRR5709838 EPTB	1.2.1	SRR6480486 EPTB	4.1.2.1
SRR5709854 EPTB	1.2.1	SRR6480489 EPTB	4.1.2.1
SRR5709856 EPTB	1.1.1	SRR6480508 EPTB	4.1.2.1
SRR5709857 EPTB	1.1.1	SRR6480585 EPTB	4.1.2.1
SRR5709858 EPTB	1.1.1	SRR6480609 EPTB	4.1.1.3
SRR5709861 EPTB	1.2.1	ERR1950087 EPTB	4.8
SRR5709975 EPTB	1.2.1	ERR2229064 EPTB	4.8
SRR5709978 EPTB	1.2.1	ERR2229066 EPTB	4.8
SRR5709979 EPTB	1.1.1	ERR2229374 EPTB	4.8
SRR5709984 EPTB	1.1.1	ERR2229774 EPTB	4.8
SRR5709995 EPTB	1.2.1	ERR2229808 EPTB	4.8
SRR5709997 EPTB	1.2.1	SRR5709973 EPTB	4.8
SRR5709998 EPTB	1.1.1	SRR6257028 EPTB	4.8
SRR5709999 EPTB	1.1.1	SRR6257086 EPTB	4.8
SRR5710001 EPTB	1.1.1	SRR6480370 EPTB	4.8
SRR5710002 EPTB	1.2.1	SRR6480452 EPTB	4.8
SRR5710003 EPTB	1.1.1	SRR6480454 EPTB	4.8
SRR5710004 EPTB	1.1.1		
SRR5710005 EPTB	1.1.1		
SRR6367399 EPTB	1.2.2		
SRR6480525 EPTB	1.2.2		
